# Telephone Triage for Emergency Patients Reduces Unnecessary Ambulance Use: A Propensity Score Analysis With Population-Based Data in Osaka City, Japan

**DOI:** 10.3389/fpubh.2022.896506

**Published:** 2022-06-28

**Authors:** Yusuke Katayama, Tetsuhisa Kitamura, Shunichiro Nakao, Hoshi Himura, Ryo Deguchi, Shunsuke Tai, Junya Tsujino, Yasumitsu Mizobata, Takeshi Shimazu, Yuko Nakagawa

**Affiliations:** ^1^Department of Traumatology and Acute Critical Medicine, Osaka University Graduate School of Medicine, Suita, Japan; ^2^Department of Environmental Medicine and Population Sciences, Department of Social and Environmental Medicine, Osaka University Graduate School of Medicine, Suita, Japan; ^3^Department of Traumatology and Critical Care Medicine, Osaka Metropolitan University Graduate School of Medicine, Osaka, Japan; ^4^Osaka Municipal Fire Department, Osaka, Japan; ^5^Osaka General Medical Center, Osaka, Japan

**Keywords:** telephone triage, ambulance, EMS, public health, propensity score

## Abstract

**Background:**

Telephone triage service in emergency care has been introduced around the world, but the impact of this service on the emergency medical service (EMS) system has not been fully revealed. The aim of this study was to evaluate the effect of telephone triage service for emergency patients on decreasing unnecessary ambulance use by analysis with propensity score (PS) matching.

**Methods:**

This study was a retrospective observational study, and the study period was the 4 years from January 2016 to December 2019. We included cases for which ambulances were dispatched from the Osaka Municipal Fire Department (OMFD). The primary outcome of this study was unnecessary ambulance use. We calculated a PS by fitting a logistic regression model to adjust for 10 variables that existed before use of the telephone triage service. To ensure the robustness of this analysis, we used not only PS matching but also a multivariable logistic regression model and regression model with PS as a covariate.

**Results:**

This study included 868,548 cases, of which 8,828 (1.0%) used telephone triage services and 859,720 (99.0%) did not use this service. Use of the telephone triage service was inversely associated with the occurrence of unnecessary ambulance use in multivariate logistic regression model (adjusted OR 0.453, 95% CI 0.405–0.506) and multivariate logistic regression model with PS as a covariate (adjusted OR 0.514, 95% CI 0.460–0.574). In the PS matching model, we also revealed same results (crude OR 0.487, 95% CI 0.425–0.588).

**Conclusions:**

In this study, we were able to statistically evaluate the effectiveness of telephone triage service already in use by the public using the statistical method with PS. As a result, it was revealed that the use of a telephone triage service was associated with a lower proportion of unnecessary ambulance use in a metropolitan area of Japan.

## Introduction

The emergency medical service (EMS) is essential social system around the world. However, unnecessary ambulance use and frequent ambulance request are problems of public health in many countries (1–3). In Japan, anyone can call for an ambulance free for charge, and the number of ambulance dispatches has been increasing in recent years (4). As a result, the time duration from ambulance call to hospital arrival is being prolonged (4), and problems such as difficulty in hospital acceptance are occurred by increasing number of patients transported by ambulance (5). This may affect ambulance dispatch to truly emergency patients such as cardiopulmonary arrest and severe trauma with shock.

A telephone triage service in emergency care has been introduced in many countries such as the United Kingdom, Canada and Australia. In these countries, telephone triage nurses use a software to assess the urgency of a patient and provide necessary services such as ambulance dispatch and sending a doctor (6–8). In Japan, a telephone triage service in emergency care was introduced in Tokyo in 2007 and Osaka in 2009. As we previously described the telephone triage service in Osaka, a telephone triage nurse assesses the urgency of the caller with software and dispatches an ambulance or directs the caller to an available medical facility based on the triage result (9). Eastwood et al. revealed that planned emergency department (ED) visits were more likely to be ED suitable than unplanned ED visits (OR 1.62; 95%CI: 1.5–1.7) (8). Another study revealed that all secondary telephone triage cases referred for emergency ambulance dispatch had transportation rates higher than all metropolitan emergency ambulance cases (82.2% vs. 71.1%) (10). However, the effect of the telephone triage service on the EMS system has not been fully revealed. If it reveals that a telephone triage service has positive effect on the EMS system, it is likely that such a service will be introduced in more countries.

Osaka city is one of the largest urban areas in Japan. A telephone triage service was introduced in 2012, and the annual number of ambulance dispatches is approximately 250,000 (11). In this study, we assessed the effect of telephone triage service for emergency patients on the decrease in unnecessary ambulance use by analysis with propensity score (PS).

## Methods

### Study Design, Setting, and Populations

This was a retrospective observational study, and the study period was 4 years from January 2016 to December 2019. Osaka city is one of the largest metropolitan areas in Japan, covering an area of 225.30 km^2^ with a population of 2.75 million (12). In Japan, the telephone triage service in emergency care and call for ambulance are public services, and anyone can use these services free of charge. In this study, the inclusion criteria were cases for which ambulances were dispatched from the Osaka Municipal Fire Department (OMFD), and the exclusion criteria were cases in which more than one patient was transported by ambulance or cases with missing data. Because we used anonymized data provided from the OMFD, the requirement of obtaining patients' informed consent was waived. This study was approved by the Ethics Committee of Osaka University Graduate School of Medicine (approval number: 16070). We wrote this manuscript based on the Strengthening the Reporting of Observational Studies in Epidemiology (STROBE) statement to assess the reporting of cohort and cross-sectional studies (13).

### Telephone Triage Service in Osaka Prefecture

The telephone triage service in Osaka prefecture has been previously described in detail (9). A telephone triage nurse evaluates the urgency of a patient's signs and symptoms using software based on the telephone triage protocol in Japan. This protocol is categorized according to 98 chief complaints (14), and the urgency of the caller is judged by selecting signs and symptoms related to the caller's chief complaints. Similar to telephone triage services in the United States, Canada, and the United Kingdom (8, 15–18), telephone triage nurses request ambulance dispatches or give a caller the information on appropriate hospitals based on the telephone triage results (19). Our software records data generated by the telephone triage such as gender, age group of the patient, duration of the telephone triage, chief complaint and associated signs, telephone triage results, and whether an ambulance was dispatched, or not.

### Main Outcome

The main outcome of this study was unnecessary ambulance use. We defined the following cases as unnecessary ambulance use: “patients refuse transport to hospital,” “there was no patient,” “ambulance call was canceled during ambulance dispatch,” “ambulance call was made as a result of mischief,” and “patient was too drunk to be transported to hospital.”

### Statistical Analysis

#### Propensity Score Matching

The purpose of this study was to evaluate the effectiveness of an intervention in which people use telephone triage service and the nurses assess the urgency of symptoms and triage callers. However, since the telephone triage service was already in existence in Osaka, Japan, we used propensity score matching as the main statistical analysis in this study. We calculated a PS by fitting a logistic regression model to adjust for the 10 variables that existed before the use of the telephone triage service. The variables used to calculate a PS were age, sex, calendar year, month, day of the week, time of day, public holiday and weekend, reason for ambulance call, administrative districts, and location of occurrence. The time of day was classified in 1-h increments. Reason for ambulance call and location of occurrence were categorized according to the ambulance record in the OMFD (20). Administrative districts were classified into 24 areas defined by Osaka city. We performed one-to-one pair matching between cases for which an ambulance was dispatched via telephone triage service or not by nearest-neighbor matching without replacement, using calipers of width equal to 0.2 of the standard deviation mean difference (SMD) of the logit of the PS. Covariate balances before and after matching were checked by comparison of SMD. A SMD of <0.1 was considered to show a negligible imbalance between the two groups (21).

#### Other Statistical Analyses

To ensure the robustness of the analysis with PS matching model, we also analyzed with a multivariable logistic regression model and a regression model with PS as a covariate. The variables entered into the multivariable logistic regression model were the 10 variables used in the calculation of the PS, and telephone triage service. In addition, we divided the age groups into children (0–14 years old), adults (15–64 years old), and the elderly (65 years old and over) and assessed them in the same way. All tests were two-tailed, and *P* values of <0.05 were considered statistically significant. All statistical analyses were performed using SPSS ver 27.0J (IBM Corp. Armonk, NY).

## Results

**Figure 1:**
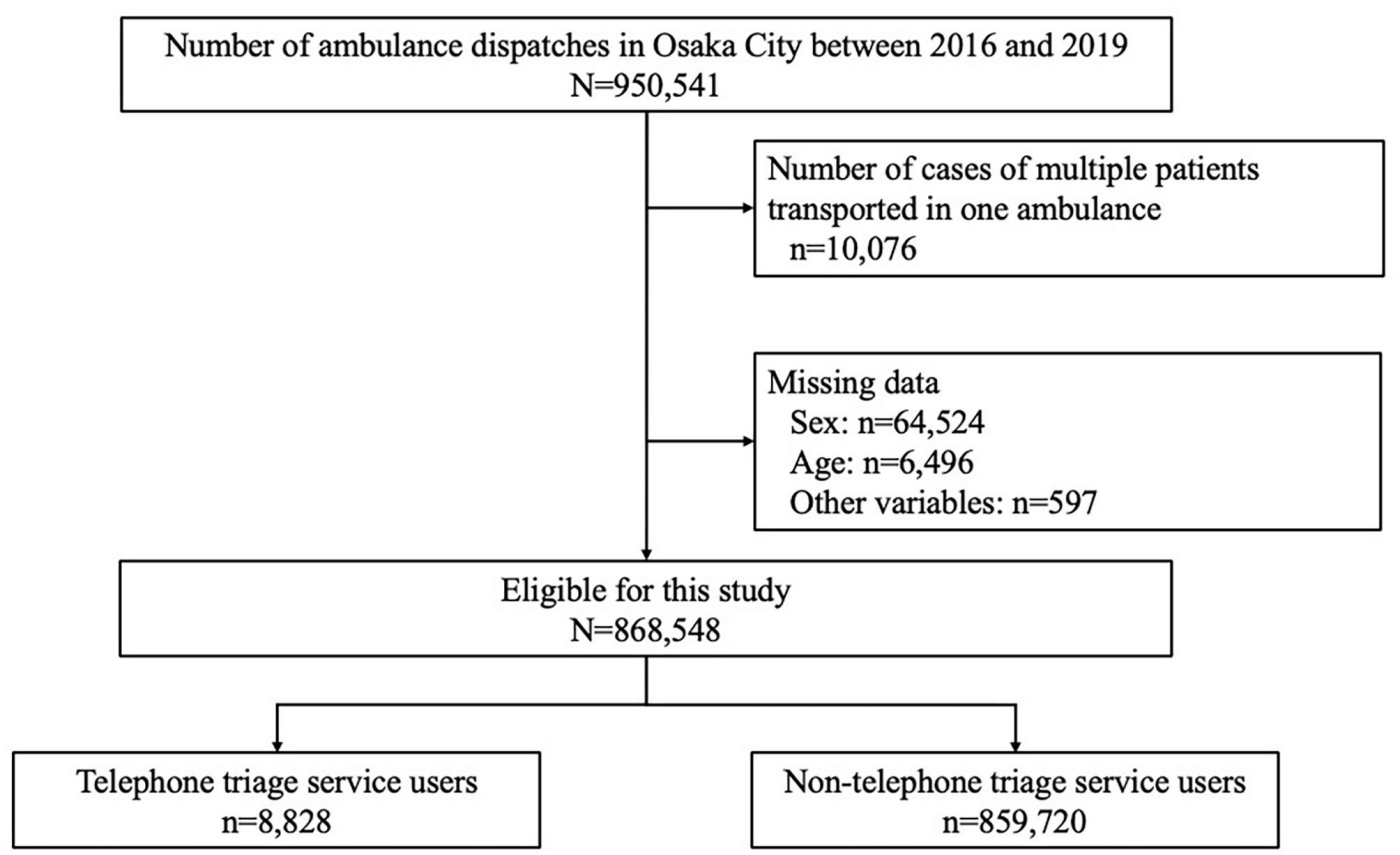
Patient flow in this study.

Figure 1 shows patient flow in this study. The number of ambulance dispatches in Osaka city was 950,541 during the study period and we included 868,548 patients in this study. Among the cases included in this study, 8,828 (1.0%) used telephone triage services and 859,720 (99.0%) did not use this service.

Table 1 shows the characteristics of the cases before and after PS matching. In all cohort before the PS matching, patients using the telephone triage service were younger, more likely to call for an ambulance due to “acute disease,” and less likely to call for an ambulance due to “traffic accident by car” and “other injury.” Regarding location of occurrence, the proportion of “home” was high, followed by that of “public space” and “road, highway and railroad” in cases using the telephone triage service. In the PS matched cohort, 8,828 cases were selected from each group, and the balances of all covariates improved between the two groups after PS matching. The area under the curve in the logistic regression model for PS calculation was 0.808.

**Table 1:** Patient characteristics among all cohorts and the propensity score-matched cohort.

	**All patients**					**Propensity score-matched patients**				
	**Telephone triage service users**		**Non-telephone triage service users**		**SMD**	**Telephone triage service users**		**Non-telephone triage service users**		**SMD**
	**(*****N*** **=** **8,828)**		**(*****N*** **=** **859,720)**			**(*****N*** **=** **8,828)**		**(*****N*** **=** **8,828)**		
Age, mean (SD)	43.4	(27.9)	58.8	(25.2)	0.579	43.4	(27.9)	44.4	(28.1)	0.036
Male, *n* (%)	4,050	(45.9%)	4,60,719	(53.6%)	0.155	4,050	(45.9%)	4,080	(45.3%)	0.012
**Year**, ***n*** **(%)**										
2016	1,984	(22.5%)	2,06,494	(24.0%)	0.037	1,984	(22.5%)	1,992	(22.6%)	0.002
2017	2,108	(23.9%)	2,10,338	(24.5%)	0.014	2,108	(23.9%)	2,163	(24.5%)	0.015
2018	2,279	(25.8%)	2,21,608	(25.8%)	0.001	2,279	(25.8%)	2,293	(26.0%)	0.004
2019	2,457	(27.8%)	2,21,280	(25.7%)	0.047	2,457	(27.8%)	2,380	(27.0%)	0.020
**Month**, ***n*** **(%)**										
January	662	(7.5%)	77,720	(9.0%)	0.056	662	(7.5%)	689	(7.8%)	0.012
February	591	(6.7%)	67,522	(7.9%)	0.045	591	(6.7%)	616	(7.0%)	0.011
March	683	(7.7%)	70,418	(8.2%)	0.017	683	(7.7%)	677	(7.7%)	0.003
April	659	(7.5%)	67,459	(7.8%)	0.014	659	(7.5%)	649	(7.4%)	0.004
May	710	(8.0%)	68,659	(8.0%)	0.002	710	(8.0%)	692	(7.8%)	0.008
June	747	(8.5%)	68,116	(7.9%)	0.020	747	(8.5%)	756	(8.6%)	0.004
July	817	(9.3%)	78,565	(9.1%)	0.004	817	(9.3%)	816	(9.2%)	0.000
August	871	(9.9%)	77,091	(9.0%)	0.031	871	(9.9%)	861	(9.8%)	0.004
September	698	(7.9%)	67,728	(7.9%)	0.001	698	(7.9%)	731	(8.3%)	0.014
October	773	(8.8%)	70,049	(8.1%)	0.022	773	(8.8%)	755	(8.6%)	0.007
November	769	(8.7%)	69,166	(8.0%)	0.024	769	(8.7%)	769	(8.7%)	0.000
December	847	(9.6%)	77,227	(9.0%)	0.021	847	(9.6%)	817	(9.3%)	0.012
**Day of the week**, ***n*** **(%)**										
Sunday	1,606	(18.2%)	1,24,529	(14.5%)	0.100	1,606	(18.2%)	1,625	(18.4%)	0.006
Monday	1,231	(13.9%)	1,27,027	(14.8%)	0.024	1,231	(13.9%)	1,247	(14.1%)	0.005
Tuesday	1,205	(13.6%)	1,20,232	(14.0%)	0.010	1,205	(13.6%)	1,226	(13.9%)	0.007
Wednesday	1,131	(12.8%)	1,17,324	(13.6%)	0.025	1,131	(12.8%)	1,049	(11.9%)	0.028
Thursday	1,238	(14.0%)	1,19,240	(13.9%)	0.004	1,238	(14.0%)	1,302	(14.7%)	0.021
Friday	1,114	(12.6%)	1,24,556	(14.5%)	0.055	1,114	(12.6%)	1,082	(12.3%)	0.011
Saturday	1,303	(14.8%)	1,26,812	(14.8%)	0.000	1,303	(14.8%)	1,297	(14.7%)	0.002
Weekend and holiday, *n* (%)	3,368	(38.2%)	2,87,380	(33.4%)	0.099	3,368	(38.2%)	3,372	(38.2%)	0.001
**Time of day**, ***n*** **(%)**										
0:00–0:59	450	(5.1%)	28,110	(3.3%)	0.091	450	(5.1%)	454	(5.1%)	0.002
1:00–1:59	372	(4.2%)	23,342	(2.7%)	0.082	372	(4.2%)	368	(4.2%)	0.002
2:00–2:59	313	(3.5%)	20,045	(2.3%)	0.072	313	(3.5%)	337	(3.8%)	0.014
3:00–3:59	252	(2.9%)	17,869	(2.1%)	0.050	252	(2.9%)	269	(3.0%)	0.011
4:00–4:59	259	(2.9%)	16,768	(2.0%)	0.064	259	(2.9%)	242	(2.7%)	0.012
5:00–5:59	236	(2.7%)	18,046	(2.1%)	0.038	236	(2.7%)	256	(2.9%)	0.014
6:00–6:59	263	(3.0%)	21,590	(2.5%)	0.029	263	(3.0%)	288	(3.3%)	0.016
7:00–7:59	315	(3.6%)	27,742	(3.2%)	0.019	315	(3.6%)	317	(3.6%)	0.001
8:00–8:59	329	(3.7%)	38,031	(4.4%)	0.035	329	(3.7%)	348	(3.9%)	0.011
9:00–9:59	308	(3.5%)	47,811	(5.6%)	0.100	308	(3.5%)	301	(3.4%)	0.004
10:00–10:59	302	(3.4%)	48,772	(5.7%)	0.108	302	(3.4%)	279	(3.2%)	0.015
11:00–11:59	266	(3.0%)	46,558	(5.4%)	0.120	266	(3.0%)	281	(3.2%)	0.010
12:00–12:59	271	(3.1%)	45,401	(5.3%)	0.111	271	(3.1%)	272	(3.1%)	0.001
13:00–13:59	319	(3.6%)	45,249	(5.3%)	0.080	319	(3.6%)	318	(3.6%)	0.001
14:00–14:59	341	(3.9%)	42,737	(5.0%)	0.054	341	(3.9%)	323	(3.7%)	0.011
15:00–15:59	290	(3.3%)	42,179	(4.9%)	0.082	290	(3.3%)	301	(3.4%)	0.007
16:00–16:59	350	(4.0%)	42,785	(5.0%)	0.049	350	(4.0%)	366	(4.1%)	0.009
17:00–17:59	371	(4.2%)	45,166	(5.3%)	0.050	371	(4.2%)	328	(3.7%)	0.025
18:00–18:59	427	(4.8%)	45,268	(5.3%)	0.020	427	(4.8%)	451	(5.1%)	0.013
19:00–19:59	570	(6.5%)	43,637	(5.1%)	0.059	570	(6.5%)	567	(6.4%)	0.001
20:00–20:59	618	(7.0%)	42,163	(4.9%)	0.089	618	(7.0%)	594	(6.7%)	0.011
21:00–21:59	568	(6.4%)	39,908	(4.6%)	0.078	568	(6.4%)	556	(6.3%)	0.006
22:00–22:59	553	(6.3%)	36,890	(4.3%)	0.088	553	(6.3%)	532	(6.0%)	0.010
23:00–23:59	485	(5.5%)	33,553	(3.9%)	0.075	485	(5.5%)	480	(5.4%)	0.002
**Reason for ambulance call**										
Fire accident	3	(0.0%)	390	(0.0%)	0.006	3	(0.0%)	0	(0%)	0.026
Natural disaster	1	(0.0%)	197	(0.0%)	0.009	1	(0.0%)	0	(0%)	0.015
Water accident	0	(0%)	182	(0.0%)	0.021	0	(0%)	0	(0%)	-
Traffic accident by car	42	(0.5%)	54,089	(6.3%)	0.326	42	(0.5%)	58	(0.7%)	0.024
Traffic accident by ship	0	(0%)	2	(0.0%)	0.002	0	(0%)	0	(0%)	-
Traffic accident by aircraft	0	(0%)	3	(0.0%)	0.003	0	(0%)	0	(0%)	-
Injury due to industrial accident	17	(0.2%)	5,883	(0.7%)	0.074	17	(0.2%)	11	(0.1%)	0.017
Poisoning and acute disease due to industrial accident	1	(0.0%)	192	(0.0%)	0.008	1	(0.0%)	1	(0.0%)	0.000
Acute disease and injury during sports	19	(0.2%)	3,770	(0.4%)	0.039	19	(0.2%)	9	(0.1%)	0.028
Acute disease and injury while watching sports	0	(0%)	104	(0.0%)	0.016	0	(0%)	0	(0%)	-
Asphyxia	106	(1.2%)	3,205	(0.4%)	0.094	106	(1.2%)	87	(1.0%)	0.021
Gas poisoning not due to industrial accident and self-injury	1	(0.0%)	62	(0.0%)	0.004	1	(0.0%)	0	(0%)	0.015
Other injury	692	(7.8%)	1,34,762	(15.7%)	0.245	692	(7.8%)	708	(8.0%)	0.007
Assault	14	(0.2%)	8,968	(1.0%)	0.115	14	(0.2%)	15	(0.2%)	0.003
Self-induced drug abuse and gas poisoning	61	(0.7%)	4,216	(0.5%)	0.026	61	(0.7%)	64	(0.7%)	0.004
Self-induced injury	6	(0.1%)	3,241	(0.4%)	0.066	6	(0.1%)	9	(0.1%)	0.012
Acute disease	7,729	(87.6%)	5,82,349	(67.7%)	0.490	7,729	(87.6%)	7,690	(87.1%)	0.013
Gynecological disease including childbirth	136	(1.5%)	6,912	(0.8%)	0.068	136	(1.5%)	142	(1.6%)	0.005
Inter-hospital transfer	0	(0%)	50,844	(5.9%)	0.355	0	(0%)	33	(0.4%)	0.087
Other	0	(0%)	349	(0.0%)	0.028	0	(0%)	1	(0.0%)	0.015
**Location of occurrence**										
Home	7,951	(90.1%)	4,52,877	(52.7%)	0.908	7,951	(90.1%)	7,951	(90.1%)	0.000
Work place	184	(2.1%)	22,817	(2.7%)	0.037	184	(2.1%)	166	(1.9%)	0.015
Public place	371	(4.2%)	2,19,016	(25.5%)	0.627	371	(4.2%)	373	(4.2%)	0.001
Public transportation	14	(0.2%)	5,569	(0.6%)	0.077	14	(0.2%)	17	(0.2%)	0.008
Road, highway and railroad	244	(2.8%)	1,47,079	(17.1%)	0.494	244	(2.8%)	248	(2.8%)	0.003
Sea, pools and rivers	0	(0%)	364	(0.0%)	0.029	0	(0%)	0	(0%)	-
Other indoor areas	12	(0.1%)	1,914	(0.2%)	0.020	12	(0.1%)	10	(0.1%)	0.006
Other outdoor areas	52	(0.6%)	10,084	(1.2%)	0.063	52	(0.6%)	63	(0.7%)	0.015
**Area**										
Kita-ku	554	(6.3%)	63,250	(7.4%)	0.043	554	(6.3%)	551	(6.2%)	0.001
Miyakojima-ku	400	(4.5%)	28,896	(3.4%)	0.060	400	(4.5%)	375	(4.2%)	0.014
Fukushima-ku	211	(2.4%)	17,809	(2.1%)	0.022	211	(2.4%)	221	(2.5%)	0.007
Konohana-ku	176	(2.0%)	21,483	(2.5%)	0.034	176	(2.0%)	196	(2.2%)	0.016
Chuo-ku	480	(5.4%)	56,022	(6.5%)	0.046	480	(5.4%)	482	(5.5%)	0.001
Nishi-ku	329	(3.7%)	27,272	(3.2%)	0.030	329	(3.7%)	306	(3.5%)	0.014
Minato-ku	219	(2.5%)	24,726	(2.9%)	0.024	219	(2.5%)	205	(2.3%)	0.010
Taisho-ku	162	(1.8%)	20,269	(2.4%)	0.036	162	(1.8%)	177	(2.0%)	0.012
Tennnoji-ku	285	(3.2%)	23,565	(2.7%)	0.029	285	(3.2%)	266	(3.0%)	0.012
Naniwa-ku	277	(3.1%)	30,694	(3.6%)	0.024	277	(3.1%)	292	(3.3%)	0.010
Nishiyodogawa-ku	251	(2.8%)	26,474	(3.1%)	0.014	251	(2.8%)	261	(3.0%)	0.007
Yodogawa-ku	557	(6.3%)	50,467	(5.9%)	0.018	557	(6.3%)	554	(6.3%)	0.001
Higashiyodogawa-ku	480	(5.4%)	45,942	(5.3%)	0.004	480	(5.4%)	481	(5.4%)	0.000
Higashinari-ku	289	(3.3%)	21,231	(2.5%)	0.048	289	(3.3%)	276	(3.1%)	0.008
Ikuno-ku	343	(3.9%)	38,807	(4.5%)	0.031	343	(3.9%)	316	(3.6%)	0.016
Asahi-ku	265	(3.0%)	22,768	(2.6%)	0.021	265	(3.0%)	277	(3.1%)	0.008
Joto-ku	519	(5.9%)	39,133	(4.6%)	0.060	519	(5.9%)	544	(6.2%)	0.012
Tsurumi-ku	323	(3.7%)	25,075	(2.9%)	0.042	323	(3.7%)	317	(3.6%)	0.004
Abeno-ku	402	(4.6%)	28,112	(3.3%)	0.066	402	(4.6%)	396	(4.5%)	0.003
Suminoe-ku	436	(4.9%)	38,658	(4.5%)	0.021	436	(4.9%)	413	(4.7%)	0.012
Sumiyoshi-ku	497	(5.6%)	41,990	(4.9%)	0.033	497	(5.6%)	524	(5.9%)	0.013
Higashisumiyoshi-ku	433	(4.9%)	36,802	(4.3%)	0.030	433	(4.9%)	427	(4.8%)	0.003
Hirano-ku	654	(7.4%)	56,502	(6.6%)	0.033	654	(7.4%)	675	(7.6%)	0.009
Nishinari-ku	286	(3.2%)	73,558	(8.6%)	0.227	286	(3.2%)	296	(3.4%)	0.006
Outside Osaka City	0	(0%)	215	(0.0%)	0.022	0	(0%)	0	(0%)	-

*EMS represents emergency medical service. SMD, standardized mean difference; SD, standard deviation; IQR, interquartile range*.

Table 2 shows the proportion of unnecessary ambulance use in all cohort and the PS-matched cohort. The number of unnecessary ambulance uses was 66,100 (7.6%) in all cohort. Of them, 330 patients (3.7%) used the telephone triage service and 65,770 patients (7.7%) did not. In the PS-matched cohort, the number of unnecessary ambulance uses was 982 (5.6%), in which 330 (3.7%) patients used the telephone triage service and 652 (7.7%) did not use the service. The use of the telephone triage service was inversely associated with the occurrence of unnecessary ambulance use in a PS matching model (crude OR 0.487, 95% CI 0.425–0.588). And, we also revealed the same results in a univariate logistic regression model (crude odds ratio [OR] 0.469, 95% confidence interval [CI] 0.420–0.523), multivariate logistic regression model (adjusted OR 0.453, 95% CI 0.405–0.506), multivariate logistic regression model with PS as a covariate (adjusted OR 0.514, 95% CI 0.460–0.574).

**Table 2:** Unnecessary ambulance use with or without telephone triage service.

	**Total**		**Telephone triage service used**		**Telephone triage service not used**		**Crude OR (95% CI)**		**Adjusted OR (95% CI)**	
All patients	(*N* = 868,548)		(*N* = 8,828)		(*N* = 859,720)					
Unnecessary ambulance use	66,100	(7.6%)	330	(3.7%)	65,770	(7.7%)				
Univariate logistic regression model							0.469	(0.420–0.523)	-	-
Multivariate logistic regression model*							-	-	0.453	(0.405–0.506)
Regression model with propensity score as covariate							-	-	0.514	(0.460–0.574)
Propensity score-matched patients	(*N* = 17,656)		(*N* = 8,828)		(*N* = 8828)					
Unnecessary ambulance use	982	(5.6%)	330	(3.7%)	652	(7.4%)	0.487	(0.425–0.588)	-	-

OR represents odds ratio; CI, confidence interval. ORs were calculated for patients with vs. without telephone triage service. *Adjusted for age, sex, calendar year, month, day of the week, time zone, holiday including weekend, reason for ambulance call, administrative district, and accident location.

Table 3 shows the proportion of unnecessary ambulance use in all cohort and PS-matched cohort among children. The proportions of unnecessary ambulance use were 3.3% (58/1,768) among the patients using the telephone triage service and 4.0% (2,103/53,097) among those not using the service. The crude OR was 0.725 (95% CI 0.513–1.024) in this PS-matched cohort.

**Table 3:** Unnecessary ambulance use with or without telephone triage service among children.

	**Total**		**Telephone triage service used**		**Telephone triage service not used**		**Crude OR (95% CI)**		**Adjusted OR (95% CI)**	
All patients	(*N* = 54,865)		(*N* = 1,768)		(*N* = 53,097)					
Unnecessary ambulance use	2,161	(3.9%)	58	(3.3%)	2,103	(4.0%)				
Univariate logistic regression model							0.822	(0.631–1.072)	-	-
Multivariate logistic regression model*							-	-	0.760	(0.581–0.995)
Regression model with propensity score as covariate							-	-	0.782	(0.599–1.022)
Propensity score-matched patients	(N = 3,536)		(N = 1,768)		(N = 1,768)					
Unnecessary ambulance use	137	(3.9%)	58	(3.3%)	79	(4.5%)	0.725	(0.513–1.024)	-	-

OR represents odds ratio; CI, confidence interval. ORs were calculated for patients with vs. without telephone triage service. *Adjusted for age, sex, calendar year, month, day of the week, time zone, holiday including weekend, reason for ambulance call, administrative district, and accident location.

Table 4 shows the proportion of unnecessary ambulance use in all cohort and PS-matched cohort among adults. The proportions of unnecessary ambulance use were 4.4% (198/4,468) among the patients using the telephone triage service and 11.1% (40,084/360,255) among those not using it. The crude OR was 0.410 (95% CI 0.345–0.487) in this PS-matched cohort.

**Table 4:** Unnecessary ambulance use with or without telephone triage service among adults.

	**Total**		**Telephone triage service used**		**Telephone triage service not used**		**Crude OR (95% CI)**		**Adjusted OR (95% CI)**	
All patients	(*N* = 364,723)		(*N* = 4,468)		(*N* = 360,255)					
Unnecessary ambulance use	40,282	(11.0%)	198	(4.4%)	40,084	(11.1%)				
Univariate logistic regression model							0.370	(0.321–0.427)	-	-
Multivariate logistic regression model*							-	-	0.393	(0.340–0.455)
Regression model with propensity score as covariate							-	-	0.428	(0.371–0.494)
Propensity score-matched patients	(*N* = 8,936)		(*N* = 4,468)		(*N* = 4,468)					
Unnecessary ambulance use	652	(7.3%)	198	(4.4%)	454	(10.2%)	0.410	(0.345–0.487)	-	-

OR represents odds ratio; CI, confidence interval. ORs were calculated for patients with vs. without telephone triage service. *Adjusted for age, sex, calendar year, month, day of the week, time zone, holiday including weekend, reason for ambulance call, administrative district, and accident location.

Table 5 shows the proportion of unnecessary ambulance use in the total cohort and PS-matched cohort among the elderly. The proportions of unnecessary ambulance use were 2.9% (74/2,592) in the patients using the telephone triage service and 5.3% (23,583/446,368) in those not using the service. The crude OR was 0.639 (95% CI 0.474–0.860) in this PS-matched cohort.

**Table 5:** Unnecessary ambulance use with or without telephone triage service among the elderly.

	**Total**		**Telephone triage service used**		**Telephone triage service not used**		**Crude OR (95% CI)**		**Adjusted OR (95% CI)**	
All patients	(*N* = 448,960)		(N = 2,592)		(*N* = 446,368)					
Unnecessary ambulance use	23,657	(5.3%)	74	(2.9%)	23,583	(5.3%)				
Univariate logistic regression model							0.527	(0.418–0.664)	-	-
Multivariate logistic regression model*							-	-	0.546	(0.432–0.689)
Regression model with propensity score as covariate							-	-	0.585	(0.464–0.737)
Propensity score-matched patients	(*N* = 5,182)		(*N* = 2,591)		(*N* = 2,591)					
Unnecessary ambulance use	188	(3.6%)	74	(2.9%)	114	(4.4%)	0.639	(0.474–0.860)	-	-

*OR represents odds ratio; CI, confidence interval*.

*ORs were calculated for patients with vs. without telephone triage service*.

*Adjusted for age, sex, calendar year, month, day of the week, time zone, holiday including weekend, reason for ambulance call, administrative district, and accident location.

## Discussion

In this study, we were able to statistically evaluate the effectiveness of telephone triage service already in use by the public using the statistical method with PS. As a result, it was revealed that the use of a telephone triage service was associated with a lower proportion of unnecessary ambulance use in a metropolitan area of Japan. In subgroup analysis by age group, although the telephone triage service was associated with a lower proportion of unnecessary ambulance use in adults and the elderly, the proportion of unnecessary ambulance use tended to be lower, but not statistically significantly so, in children. To the best of our knowledge, there is no study using population-based data to assess the impact of a telephone triage service for emergency patients on the EMS system, and the findings of this study may help to improve EMS systems around the world.

First, we found that the proportion of unnecessary ambulance use was lower in cases for which an ambulance was dispatched via telephone triage service than in cases without telephone triage service. In Japan, because calling for an ambulance is free of charge, it may be called for even in less urgent cases. Therefore, people may be calling for an ambulance even when they do not necessarily need it. Several previous studies have shown the effect of telephone triage in reducing emergency department visits and same-day visits to health care facilities (22–24). In an observational study by Hogenbirk et al. in Canada, telephone triage advice reduced caller intention to visit the emergency department, and the effect appears to be stronger in communities with a weak or no transport link than in urban areas (24). In contrast, Richards et al. reported that telephone triage in primary care increased both the workload of nurses and the number of out-of-hours visits and ambulance dispatches for accidents (25). Furthermore, Doctor et al. found that one-third of patients who visited emergency departments after telephone triage did not require referral to the emergency department (26). Thus, the effect of telephone triage services is controversial and may be related to differences in health care systems in each country. A previous study by Turbitt and Freed in Victoria, Australia reported a 20% awareness of telephone triage services among patients who visited emergency departments with non-urgent children (27). To make telephone triage service work, it is important to increase public awareness and to spread information on the effectiveness of the service. Thus, the present study is useful because we revealed the impact of telephone triage services on an EMS system. We showed that only 1% of ambulances were dispatched via telephone triage. In Japan, anyone can call 1-1-9 for free access to an ambulance. In other countries such as Australia, the telephone triage service is used to triage the call and then transfer it to the ambulance dispatch center (8, 10). To make telephone triage service more effective, it may be necessary not only to educate the public but also to change the social system for calling for an ambulance.

The use of a telephone triage service in the present study was associated with a lower rate of unnecessary ambulance use in adults and the elderly, but the rate was not statistically significantly lower in children. Several studies have reported higher rates of ambulance transport among the elderly visiting a emergency department (28, 29). Durant and Fahimi reported that older age, Medicare, Medicaid, and nighttime were associated with less urgent ambulance use (3). Elderly people with health anxiety may call for an ambulance when they are worried about their health, even if it is not an emergency situation. Telephone triage services relieve the anxiety of such callers by determining the urgency of their symptoms and conditions using software with a triage protocol. As a result, few callers with such health concerns called for an ambulance via the telephone triage service, and the proportion of unnecessary ambulance use was probably lower among them. Thus, the telephone triage service may help to make the EMS system more efficient. However, the effect of the telephone triage service was smaller in children than in adults and the elderly, and the reason for this result was unclear. In many cases, parents or guardians are the ones who call for an ambulance for a suddenly sick or injured child, and when they do, they may strongly prefer to transport the child to a medical facility and see a doctor. This may explain why the proportion of unnecessary ambulance use was low even in cases for which the telephone triage service was not used, and why the effect of the service was not statistically significant in pediatric patients. In the PS-matched cohort, the proportion of unnecessary ambulance use was 7.4% in cases without telephone triage service vs. 3.7% in cases with telephone triage service. In a previous study, the cost of an ambulance call in Japan was estimated to be 45,000 yen (400 dollars) (30). If the proportion of unnecessary ambulance use was to be reduced to 3.7% by the use of telephone triage service, this would result in an annual saving of 407,925,000 yen (US$3.7 million) in Osaka city. As the annual cost of telephone triage service in Osaka city is ~200,000,000 yen (US$1.8 million) (31), this would result in a reduction of ~200,000,000 yen (US$1.8 million). In this way, the telephone triage service is an essential tool in the EMS system as it may reduce the cost of government through effective dispatching of ambulances.

In recent years, infrastructures of centralized personal health records (PHR) are being built using blockchain technology (32). If such a PHR infrastructure can be built and used for telephone triage, it would lead to the realization of tailor-made telephone triage service based on patient's factors such as past medical history and medication history. In addition, it may also be possible to evaluate the outcome of cases in which patients are not urgent and visit a clinic on their own without ambulance transport as a result of telephone triage, as well as to track long-term prognosis such as return to work after rehabilitation. Thus, as the PHR infrastructure is built up, there will be scope for further enhancement of the telephone triage service in the future.

### Limitations

This study has some limitations. First, we did not assess the patient's family structure or the relationship between the patient and the person who called for an ambulance, such as a family member, colleague, or bystander. Second, we did not assess the impact of using the telephone triage service on patient outcomes. We are currently studying this and will publish our findings in the future. Third, the outcomes were unknown in the cases for which an ambulance was not dispatched as a result of telephone triage. In addition, this study did not include a detailed cost analysis, but we plan to evaluate quality-adjusted life years and incremental cost-effectiveness ratios. Finally, as this is an observational study, there may be unknown confounding factors.

## Conclusion

In observational studies, bias of background factors is a problem when comparing outcomes between groups, and this study made an effort to minimize those biases as much as possible by using the statistical analysis with PS. As a result, we found that the use of the telephone triage service in Osaka city reduced unnecessary ambulance use, especially among adults and the elderly in this study.

## Data Availability Statement

The data analyzed in this study is subject to the following licenses/restrictions: The data that support the findings of this study are available from the OMFD but restrictions apply to the availability of these data, which were used under the personal information protection ordinance of Osaka City, and so are not publicly available. Requests to access these datasets should be directed to Osaka Municipal Fire Department (in Japanese) https://www.city.osaka.lg.jp/shobo/page/0000052526.html.

## Ethics Statement

This study was approved by the Ethics Committees of the Osaka Graduate School of Medicine (Approval No: 16070). The requirement to obtain patient consent to participate was waived because the data were anonymized.

## Author Contributions

YK analyzed the data and wrote the first draft of this manuscript. TK reviewed all statistical analyses and critically revised this manuscript. SN, HH, and RD interpreted the data and critically revised this manuscript. ST and JT did data cleaning and provided the data for analysis. YM, TS, and YN supervised the interpretation of the data and critically revised this manuscript. All authors read and approved the final manuscript.

## Funding

This study was supported by the Fire and Disaster Prevention Technologies Program (Grant No: JPJ000255).

## Conflict of Interest

The authors declare that the research was conducted in the absence of any commercial or financial relationships that could be construed as a potential conflict of interest.

## Publisher's Note

All claims expressed in this article are solely those of the authors and do not necessarily represent those of their affiliated organizations, or those of the publisher, the editors and the reviewers. Any product that may be evaluated in this article, or claim that may be made by its manufacturer, is not guaranteed or endorsed by the publisher.

## Abbreviations

CI: confidence interval
EMS: emergency medical service
OMFD: Osaka Municipal Fire Department
OR: odds ratio
PS: propensity score
SMD: standardized mean difference
STROBE: Strengthening the Reporting of Observational studies in Epidemiology.
